# Effect of Nonthermal Plasma on Shear Bond Strength of Translucent Zirconia in Layering Ceramic

**DOI:** 10.1155/2023/6639030

**Published:** 2023-05-15

**Authors:** Dorsa Seyedi, Sara Valizadeh, Safoura Ghodsi, Kimia Salimi, Faezeh Atri

**Affiliations:** ^1^Department of Prosthodontics, School of Dentistry, Tehran University of Medical Sciences, Tehran, Iran; ^2^Department of Operative Dentistry, Dental Research Center, School of Dentistry, Dentistry Research Institute, Tehran University of Medical Sciences, Tehran, Iran; ^3^Department of Prosthodontics, Dental Research Center, School of Dentistry, Dentistry Research Institute, Tehran University of Medical Sciences, Tehran, Iran

## Abstract

**Background:**

Today, various methods are used to increase the bond strength of zirconia in layering ceramics. This study evaluated the effects of nonthermal argon plasma on zirconia shear bond strength to layering porcelain. *Materials and Method*. In this experimental study, 42 square blocks of zirconia were prepared and randomly divided into three groups (*n* = 14) according to the applying surface treatment: (1) the control group (without any surface treatment), (2) the plasma-treated group with argon nonthermal plasma, and (3) the air abrasion group with 50 *µ*m Al_2_O_3_ particles. All samples were layered with porcelain. One sample from each group was evaluated by electron microscopy (SEM) to examine the cross-sectional area of the zirconia–ceramic bond. The rest of the specimens were subjected to thermocycling with 5,000 baths to imitate the aging process in the mouth and then were tested for shear bond strength. The failure pattern of the samples was examined by stereomicroscope. Bond strength data were analyzed by one-way ANOVA test in three groups and Tamhane post hoc test in pairs. The significance level of *p*-value was considered 0.05.

**Results:**

The shear bond strength of the plasma-treated group was significantly higher than the control group (*p* = 0.032) but the shear bond strength between the sandblast and the plasma-treated group was not significantly different (*p* = 0.656). The shear bond strength between the sandblast and the control group was also not significant (*p* = 0.202). Regarding the mode of failure, failures were mostly adhesive and then mixed. Examination of the samples under SEM showed that the bond area is the thickest in the sandblast group and also the surface roughness is the highest in the sandblast group and the lowest in the control group.

**Conclusion:**

This study demonstrated that the use of nonthermal argon plasma treatment is an effective way to enhance the quality and quantity of shear bond strength between layering porcelain and zirconia.

## 1. Introduction

Zirconia, especially yttria-stabilized tetragonal zirconia polycrystal (Y-TZP), has become one of the most widely used materials in the manufacture of fixed veneers and prostheses. The outstanding properties of zirconia, such as high strength, biocompatibility, and esthetic have made it the best alternative to metal–ceramic prostheses [1, 2]. New generations of zirconia are presented in different levels of translucency (translucent, high-translucent, super-translucent, and extra-translucent) which differ in their microstructure [3]. Although high-translucent zirconia was introduced for monolithic veneers, the problem of achieving a completely tooth-like color and translucency similar to enamel still remains, therefore porcelain layering is still needed in the anterior regions for optimum esthetic [4, 5].

The chipping of ceramic veneer is one of the common failures of zirconia-based restorations. The most important factor influencing this weak bond is the large differences in the mechanical properties of these two materials [6]. Some studies have been conducted on different surface treatments to increase the bond strength of opaque zirconia to layering porcelain [7–10]. The translucent zirconia which is different from opaque zirconia in microstructure and mechanical properties, might show different bonding behavior [3].

One of the common methods used is air abrasion, which causes surface roughness [11]. One of its drawbacks is the changes in the chemical properties of the surface due to contamination with alumina particles. Some studies have shown that the zirconia air abrasion results in slight increase in the bonding rate but also increases the risk of microcracks in zirconia [12, 13]. However, studies on the use of air abrasion to enhance zirconia-to-porcelain bonding are controversial [14, 15].

Lasers are one of the recently introduced methods to increase surface roughness. The laser beam has been shown to be a relatively safe and useful device for making surface roughness. Lasers have also been used to improve the wettability of zirconia surfaces [16]. Laser generators, such as Er: YAG, CO_2_, and Nd: YAG, have been used in many studies to create surface roughness [10, 17]. Another effective laser system is Er, Cr: YSGG [18, 19]. CO_2_ laser is recommended as a suitable method for surface treatment because it creates high surface roughness and provides satisfactory shear bond strength values [20, 21]. In one study, the effectiveness of different CO_2_ laser outputs was evaluated and it was shown that high output power causes a lot of surface damage compared with low output power, but the problem of laser with low output power is the inability to cause proper surface roughness [22]. Also, the heat generated by the laser has adverse side effects on the surface of the zirconia, such as microcracks and heat-affected zones [23]. The laser can also convert the low-stability tetragonal phase in Y-TZP to a monoclinic phase [24].

One of the other alternative methods is the use of nonthermal plasma (NTP) technology, which is economically viable. NTP technology is composed of ionized gases in an unbalanced environment that produces a large number of chemically active compounds such as O_3_, OH, H_2_O_2_, and NO. These active molecules can convert inactive surfaces into active ones without affecting other physical properties of the substance. The surface energy of the material increases after exposure to NTP, making it more ready to react with new molecules. Therefore, the surface energy of zirconia can be increased, and thus its bond strength can be optimized [25]. Nonthermal or low-temperature plasma generated by oxygen, argon, or mixtures in various ratios has been shown to effectively increase the hydrophilicity and surface energy of Y-TZP [7, 26].

To the best of our knowledge, no studies have been performed on the effects of surface plasma treatment on translucent zirconia, and previous studies have been performed on tetragonal zirconia [4, 16, 24, 27–30]. The present study investigates the effect of two different surface treatment methods on the shear bond between translucent zirconia and layering porcelain in comparison to the group without surface treatment. The main hypothesis is that the shear bond strength is similar in all three groups (null hypothesis).

## 2. Materials and Methods

### 2.1. Sample Preparation

**Figure 1:**
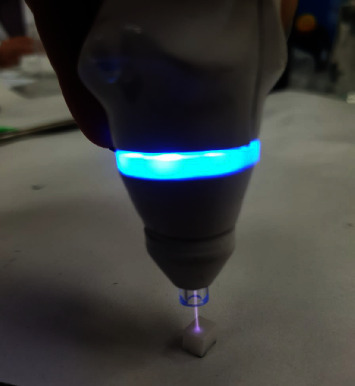
Nonthermal plasma treatment of zirconia samples.

**Figure 2:**
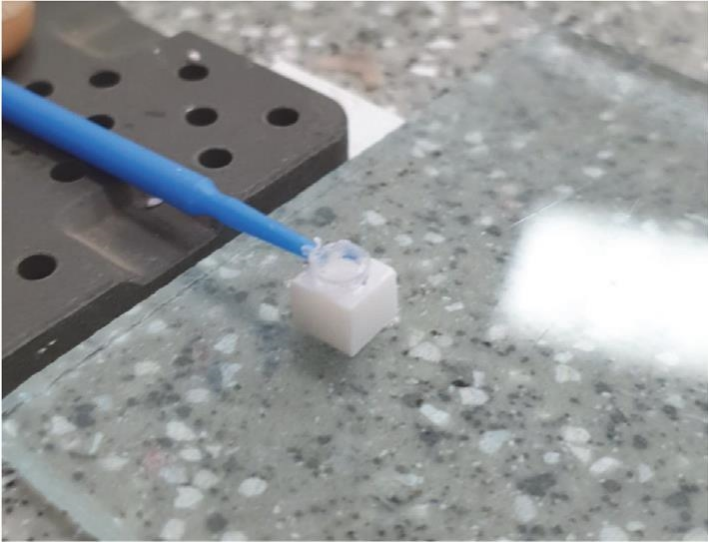
The jig designed for applying porcelain.

This in vitro study was performed on monolithic translucent zirconia (inCoris TZI mono L3, Sirona, New York City, United States). The sample size was calculated according to Bitencourt et al. [31] by using one-way ANOVA test considering *α* = 0.05 and *β* = 0.186. The least sample numbers were determined to be 13. At first, the presintered blocks were cut into 42 samples of 5 × 5 × 5 mm cubes by the use of mecatome (Presi, Eybens, France). These blocks were then sintered in Fire HTC speed furnace (Sirona, New York City, United States) for 2 hr holding time at 1,530°C. Each of the zirconia blocks was then polished with 400, 600, and 800 grits silicon carbide abrasive papers for 1 min, and at the same time water was used as a coolant to bring the surface to a suitable surface roughness. Then they were cleaned for 3 min in ultrasonic with 96% ethanol. Finally, these blocks were cleaned using distilled water [32]. These blocks were divided into three groups according to the surface treatment they received:

1. Negative control group that did not receive any surface treatment.
2. Positive control group that was sandblasted using air abrasion device with 50 *µ* aluminum oxide particles at a pressure of 1.5 bar for 10 s vertically.
3. Nonthermal plasma-treated group: In this group, the samples were irradiated from a distance of 10 mm with a nonthermal plasma machine (Medaion, Tehran, Iran) using argon gas with a flow of 3 L/min and a voltage of 4 kW in vertical radiation angle (Figure 1).
4. Ceramic veneer powder (Noritake Cerabien ZR, Kuraray, Japan) was mixed with the liquid according to the factory instructions and then placed on the zirconia by the same jig designed with a diameter of 3 mm and a height of 1 mm as shown in Figure 2.

The layered blocks were then backed just in one cycle according to the manufacturer's program with a start temperature of 600° for 1 min, and then the temperature increased at a rate of 45° per second to 930°C, and the samples were left at this temperature for 1 min. To control the height and diameter of the porcelain, all samples were checked by one technician, using the same loop to fill all the inner sides.

One sample of each group was used to examine the porcelain and zirconia interface. For this purpose, first, the samples were mounted in polyester and then cut from the middle by Mecatome (Presi, Eybens, France). Then the samples were observed by field emission scanning electron microscopy (FE-SEM) device (FEI Nova NanoSEM450, FEI, Oregon, United States) with magnifications of 250, 500, 1,000, 1,500, 3,000, 6,000, 1,2000, and 24,000 times.

Thirteen other samples from each group were first immersed in distilled water for 24 hr in an incubator (Kavoshmega, Tehran, Iran) at a temperature of 37° and underwent thermocycling to complete the polymerization process. They were subjected to a thermocycling process for 5,000 baths between 5°C and 55°C and the remaining 30 s in water and the transfer time between baths was 15 s [6].

Universal testing machine (Zwick, Berlin, Germany) was used to test the shear bond strength. First, the zirconia blocks were mounted in metal molds with self-cure acrylic. Blocks were then fixed in a special holder, and a metal blade was placed near the porcelain and zirconia interface, advancing at a speed of 1 mm/min until failure occurred [25]. The bond strength for each specimen was calculated by dividing the fracture load in Newtons by the surface area (mm^2^) to measure the strength in MPa.

**Figure 3:**
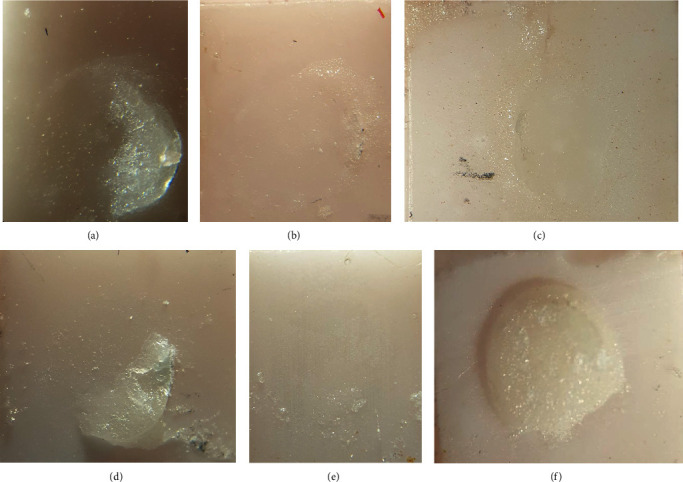
Stereomicroscope images with magnification of 500 times, mode of failure in three study groups: (a) mixed failure in plasma-prepared group, (b) adhesive failure in plasma-prepared group, (c) adhesive failure in sandblast-prepared group, (d) mixed failure in sandblast-prepared group, (e) adhesive failure in control group, and (f) cohesive failure in control group.

Failure types were examined under stereomicroscope (SMZ800, Nikon, Tokyo, Japan) and were defined as three types: (1) adhesive: in the zirconia/veneering ceramic interface, (2) cohesive: separation through the veneer ceramic material, and (3) mixed: acombination of the cohesive and adhesive types [26] (Figure 3).

The Kolmogorov–Smirnov one-sample test was used to examine the data distribution. The shear bond strength in different groups was compared using the data from the normal distribution using one-way ANOVA test and analyzed using SPSS version 22 with *p*-value = 0.05. Tamhane post hoc test was used to compare groups two by two.

## 3. Results

The Kolmogorov–Smirnov one-sample test showed in all three groups the *p*-value was greater than 0.05, and the distribution was normal. Then in the scatterplot, it was observed that one instance in each group was outdated, which we deleted.  Table 1 shows the shear bond strength (SBS) value for each experimental group. One-way ANOVA analysis showed statistically significant differences within the experimental groups (*p*-value = 0.017). Due to the significant difference between data scatter, we used Tamhane post hoc tests for pair comparison, which are less powerful. There was no significant difference between shear bond strength in the plasma-treated group and air abrasion (*p*-value = 0.656) but there was a significant difference between the plasma-treated group and the control group (*p*-value = 0.032). Also, there is no significant difference between shear bond strength in the air abrasion group and control (*p*-value = 0.202).

In terms of mode of failure, the failures were generally adhesive and then mixed. Among all samples, only one sample from the control group showed cohesive failure in porcelain bulk. Failure types are shown in Table 2.

**Figure 4:**
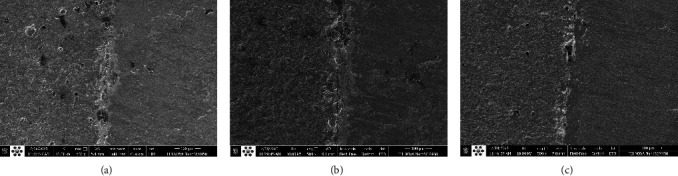
SEM images of porcelain and zirconia interfaces with magnification of 500 times in three study groups ((a) plasma-prepared group, (b) sandblast-prepared group, and (c) control group).

SEM images of the interface are shown in Figure 4. The bond area in the control group is thinner than the other two groups, slightly thicker in the plasma group, and thickest in the air abrasion group. Regarding surface roughness, in the air abrasion group, surface roughness is more than the two groups and, in the control group, surface roughness is less than the other two groups.

## 4. Discussion

The purpose of the present study was to investigate the shear bond strength of zirconia to layering porcelain in three groups of control, air abrasion, and nonthermal plasma. Based on the current findings, plasma significantly increases the shear bond strength of zirconia to layering porcelain compared with the control group, which rejects our null hypothesis that the bond strength is equal in all three groups.

Previous studies have reported various treatment methods for zirconia's surface such as grinding and air abrasion. However, their results are controversial and also there is no consensus on which approach could enhance shear bond strength values [13, 27, 33]. Furthermore, most studies were performed on tetragonal and opaque zirconia, and research on the bonding strength of translucent zirconia to layering porcelain is rare [3].

Air abrasion is the most common method for increasing the bond strength between zirconia and porcelain. Although sandblast is used to create surface roughness on zirconia and lead to micromechanical bond between porcelain and zirconia, it also produces monoclinic phase grains, which impairs the long-term strength of zirconia–porcelain [16, 34]. However, there is still controversy in the literature about its effectiveness and reliability in improving the long-term bond strength between porcelain and zirconia [28]. In the present study, 50 *µ* aluminum oxide particles were used for sandblasting because smaller aluminum oxide particles improve the bond strength without much damage to the zirconia surface and create less stress on its surface [29]. The results of the shear bond strength test showed the air abrasion group did not differ significantly from the control group.

In the present study, argon gas was used with a flow of 1 L/min for 60 s to prepare zirconia samples with nonthermal plasma [35]. The result showed that the shear bond strength in the group prepared with argon plasma was significantly higher than the control group, which was consistent with the previous studies [28, 30, 31, 36]. Plasma therapy has been reported to increase surface hydroxylation [37]. Other studies have shown that plasma therapy destroys C–C and C–H bonds and removes surface contaminations [38]. It has also been shown that increasing the surface oxygen content and its bonding to SiO_2_ of layering porcelain improves bond strength.

It should be considered that although this plasma surface decontamination may be useful to increase the bond of zirconia to porcelain, plasma-cleaned zirconia surfaces become easily recontaminated when exposed to air. In the dental laboratory, the porcelain setting stage could be delayed for a variety of reasons, recontaminating the zirconia surface and may affect the bonding of porcelain to zirconia surfaces under plasma treatment. Therefore, for the practical application of this technology, it is also important to how the prepared zirconia is stored until the porcelain application [39]. Lee et al. [36] study showed that in case of delay for layering, storing zirconia in water compared with air preserves the surface cleaned for bonding.

It must be considered that evaluation of the results of shear bond strength test has limitations compared with the clinical situation; however, it is one of the most common tests used to evaluate the bond strength between different dental materials but its results should be interpreted with caution [4].

Regarding the mode of failure, among all the samples, there was only one cohesive failure in the control group, which could be attributed to a defect the porcelain structure such as a void in layering. In both air abrasion and plasma groups, the type of mixed failure was more than adhesive failure, which could indicate an improvement in the bond strength compared with the control group [25].

SEM analysis revealed that the bond area in the plasma and sandblast group is thicker than the control group, which indicates the improvement of the bond, and also the surface violence created in the sandblast group is more than in the two groups. This finding is in line with Cardelli et al.'s [40] study, which supported a good connection and adhesion between sandblasted zirconia and layering porcelain.

The limitations of the study could be summarized as using only one gas, one type of porcelain, and lack of using thermocycling tests to imitate oral environment conditions. Therefore, we suggest that further studies focus on these modifications. Also, samples in the form of tooth-like anatomy and storage in artificial saliva can bring the results closer to the clinical conditions. The present report evaluated bond strength under ideal laboratory conditions. However, if clinicians tend to try in zirconia framework intraorally before layering, contamination with saliva [41], blood [42], bleaching agents [43], or other contaminants is probable which has been demonstrated to have a significant influence on bond strength. Therefore, also these variables should be taken into careful consideration in future clinical and laboratory tests.

## 5. Conclusion

Based on the present study, it can be concluded that nonthermal plasma treatment can be used as an effective treatment of zirconia surface to increase its bond strength in layering porcelain.

## Figures and Tables

**Table 1:** Shear bond strength (MPa) for the experimental groups (*n* = 13).

Group	Mean (MPa)	Standard deviation	Minimum (MPa)	Maximum (MPa)	Median (MPa)
Control	35.3946	18.54	24.18	45.60	40.165
Sandblast	47.2646	12.99	39.41	55.11	48.09
Plasma	52.3285	11.28	45.51	59.14	50.535

**Table 2:** Distribution of failure types in different preparation groups.

Plasma	6	0	7
Sandblast	8	0	6
Control	10	1	2

## Data Availability

Data used in this study are available from the corresponding author upon request.
